# Neurocysticercosis masquerading as a hand knob stroke

**DOI:** 10.1093/omcr/omag008

**Published:** 2026-02-24

**Authors:** Sunil Munakomi, Mandakini Parajuli

**Affiliations:** Department of Neurosurgery, Birat Medical College and teaching hospital, Biratnagar, Tankisinuwari-2, Budhiganga, Morang, Province-1, 56613, Nepal; Department of Neurosurgery, Birat Medical College and teaching hospital, Biratnagar, Tankisinuwari-2, Budhiganga, Morang, Province-1, 56613, Nepal

**Keywords:** stroke, neurology, radiology, emergency medicine

A 68-year-old patient presented with an acute onset of weakness of his right upper limb, predominantly involving his hand and wrist, for the last two days. Neurological examination revealed pure motor monoparesis with a Medical Research Council grade of 3/5, preferentially involving his hand grip and wrist extension. MRI brain ironically demonstrated features revealed a solitary lesion with scolex with perilesional edema involving the hand knob region of left motor cortex (Fig. 1). These features were highly suggestive of neurocysticercosis. Oral albendazole (400 mg twice daily) and intravenous dexamethasone (6 mg daily) were initiated as per current management guidelines. He showed drastic improvement in his motor functioning within a few days. He was discharged on albendazole and a tapering dose of oral prednisolone with advice for periodic follow-up.

**Figure 1:**
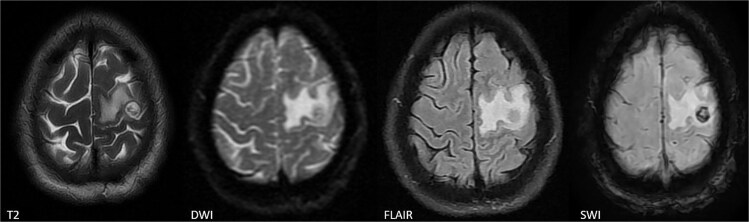
MRI sequences highly suggestive of a neurocysticercosis involving the left hand knob region.

Neurocysticercosis masquerading as hand knob stroke is a rare epiphenomenon [1, 2]. This results from the mass effect upon the affected Penfield homunculus area [2, 3]. The clinical spectrum depends on the location, number, size, and pattern of the host's immune response. Clinical examination, radio imaging, and serological tests are cornerstones for making an accurate diagnosis. In multiple NCCs, multiple stages (vesicular, colloidal, granular-nodular, and calcified) can be concurrently observed [2].

Cortical causes of monoparesis include infarction, hemiplegic migraine, seizure, multiple sclerosis, tumors, and head injuries. Radio-imaging can easily differentiate between hemorrhage, infarction, or mass lesions. Lacunar infarction and tumors do not show rapid clinical reversibility [2]. Multiple sclerosis, on the other hand, invariably shows dissemination in time and space.

There should be a high index of suspicion for neurocysticercosis among cohorts with acute-onset branchial monoparesis, especially in high-endemic zones, due to its potential for rapid reversibility [3].

The worldwide topographic pattern of endemicity of neurocysticercosis, along with a list of differential diagnoses and their salient clinical hallmarks, is provided in the Supplementary File 1  (Tables  1 and 2).

## Supplementary Material

omag008_Supplementory_File
