# Supplementary material for: Neurocysticercosis masquerading as a hand knob stroke
Source: Oxf Med Case Reports. 2026 Feb 24;2026(2):156–7. doi: 10.1093/omcr/omag008 (PMC12931322; doi:10.1093/omcr/omag008)
Supplement: omag008_Supplementory_File [file omag008_supplementory_file.pdf]

Table 1

Region	Endemicity Level	Countries/Areas	Contributing Factors
**Latin America**	High	Mexico, Guatemala, Honduras, Nicaragua, Peru, Bolivia, Ecuador, Colombia, Brazil	Poor sanitation, free-roaming pigs, undercooked pork, limited meat inspection
**Sub-Saharan Africa**	High	Tanzania, Mozambique, Zambia, Nigeria, Cameroon, Democratic Republic of Congo	Backyard pig farming, lack of sanitation, limited awareness of parasitic diseases
**South Asia**	High	India, Nepal, Bangladesh	Open defecation, pork consumption in rural areas, lack of sanitation
**East & Southeast Asia**	Moderate–High	China (rural areas), Vietnam, Indonesia, Thailand, Laos, Philippines	Ethnic food practices, poor meat inspection, traditional pig husbandry
**Middle East**	Low–Moderate	Iran, Iraq, Lebanon (sporadic cases)	Migration from endemic areas, limited but present pig farming in some regions
**Eastern Europe**	Low–Moderate	Romania, Ukraine, Serbia (sporadic foci)	Rural pig farming, improved sanitation reducing transmission
**Papua New Guinea**	Moderate–High	Highland and rural regions	Traditional pig husbandry, poor sanitation
**Caribbean**	Low–Moderate	Haiti, Dominican Republic	Poor sanitation in rural areas, pork consumption
**Industrialized Countries**	Low (Non-endemic)	USA, Canada, Australia, Western Europe (UK, Spain, France, etc.)	Imported cases via immigrants, refugees, travelers, occasional local transmission

Notes:

- **High endemicity**: Widespread local transmission with frequent clinical cases.
- **Moderate endemicity**: Localized foci with regional variation.
- **Low endemicity**: Sporadic or imported cases with minimal local transmission.
- **Non-endemic**: No local transmission; all cases are imported.

Table 2

Etiology	Type	Notes
Ischemic Stroke	Central	Most common; often affects hand or face area
Brain Tumor (e.g., metastasis)	Central	Subacute onset; may mimic stroke
Subdural Hematoma	Central	Especially in those with falls or anticoagulation

Etiology	Type	Notes
Cervical Spondylotic Myelopathy	Central	Often progressive; may include sensory signs
Radial/Ulnar Nerve Palsy	Peripheral	Often from compression or trauma
ALS	Mixed (Central & Peripheral)	May start focally; progressive
Neurocysticercosis	Central	Rare but should be considered in endemic regions
